# Recruitment of Histone Deacetylase 3 to the Interferon-A Gene Promoters Attenuates Interferon Expression

**DOI:** 10.1371/journal.pone.0038336

**Published:** 2012-06-07

**Authors:** Pierre Génin, Rongtuan Lin, John Hiscott, Ahmet Civas

**Affiliations:** 1 Centre National de la Recherche Scientifique - FRE3235, Paris Descartes University, Paris, France; 2 Lady Davis Institute-Jewish General Hospital, McGill University, Montreal, Canada; 3 Vaccine & Gene Therapy Institute of Florida, Port St. Lucie, Florida, United States of America; University of Ottawa, Canada

## Abstract

**Background:**

Induction of Type I Interferon (IFN) genes constitutes an essential step leading to innate immune responses during virus infection. Sendai virus (SeV) infection of B lymphoid Namalwa cells transiently induces the transcriptional expression of multiple IFN-A genes. Although transcriptional activation of IFN-A genes has been extensively studied, the mechanism responsible for the attenuation of their expression remains to be determined.

**Principal Findings:**

In this study, we demonstrate that virus infection of Namalwa cells induces transient recruitment of HDAC3 (histone deacetylase 3) to IFN-A promoters. Analysis of chromatin-protein association by Chip-QPCR demonstrated that recruitment of interferon regulatory factor (IRF)3 and IRF7, as well as TBP correlated with enhanced histone H3K9 and H3K14 acetylation, whereas recruitment of HDAC3 correlated with inhibition of histone H3K9/K14 acetylation, removal of IRF7 and TATA-binding protein (TBP) from IFN-A promoters and inhibition of virus-induced IFN-A gene transcription. Additionally, HDAC3 overexpression reduced, and HDAC3 depletion by siRNA enhanced IFN-A gene expression. Furthermore, activation of IRF7 enhanced histone H3K9/K14 acetylation and IFN-A gene expression, whereas activation of both IRF7 and IRF3 led to recruitment of HDAC3 to the IFN-A gene promoters, resulting in impaired histone H3K9 acetylation and attenuation of IFN-A gene transcription.

**Conclusion:**

Altogether these data indicate that reversal of histone H3K9/K14 acetylation by HDAC3 is required for attenuation of IFN-A gene transcription during viral infection.

## Introduction

Knowledge of the host signaling pathways and post-translational modifications that sense and respond to virus infection has considerably progressed in recent years [1], [2], [3], [4]. These studies demonstrate that the regulation of virus-induced gene transcription constitutes an essential step in the control of host innate antiviral responses. Signals emanating from RIG-I-like helicases (RLHs) and Toll-like receptors (TLRs) following recognition of viral ligands, converge on interferon regulatory factors IRF3 and IRF7 to regulate the induction of type I IFN genes [5], [6], [7], [8], [9]. In a majority of cell types – epithelial, fibroblastic and myeloid dendritic cells – virus-induced IFN-A gene expression is transient and requires both IRF3 and IRF7 activities, whereas activation of IRF7 by TLR7/9-mediated signaling pathways is critical for rapid and massive induction of IFN-A genes in plasmacytoid dendritic cells [10], [11], [12], [13]. IRF3 and IRF7 also participate together with NF-κB and ATF2/c-Jun in the regulation of virus-induced IFN-B gene expression [14], [15]. Once secreted from infected cells, different IFN-α subtypes and IFN-β interact with the same cell surface receptor and initiate antiviral, antitumoral or apoptotic responses by specifically inducing the expression of a large group of IFN-stimulated genes (ISGs) involved in innate and adaptive immunity [16], [17]. The powerful antiviral and immune modulatory effects of IFNs must be tightly regulated to protect against potentially harmful physiological effects that in some circumstances lead to autoimmune diseases [18], [19], [20], [21]. Host-mediated inhibition of IFN gene expression is a powerful mechanism used by the organism to restrain these negative effects.

Different control mechanisms have been proposed to explain host-mediated inhibition of type I IFN gene expression. Post-inductional repression of transcription, resulting in a rapid decrease in mRNA levels is an effective mechanism observed with IFN-B [22], [23]. In this case, PRDI-BF1 (positive regulatory domain I-binding factor-1) inhibits transcription by recruiting a co-repressor complex, whereas IRF2 prevents recruitment of the CBP/p300 co-activator and RNA Pol II complex [24], [25], [26]. Both factors interact with the IRF sites of the IFN-B promoter; the presence of highly conserved IRF sites within IFN-A promoters suggests that similar post-induction mechanisms may also regulate IFN-A gene expression. Ubiquitin-mediated degradation of IRF3 and IRF7 constitutes another important mechanism to limit virus-induced type I IFN production [27], [28], [29]. Other negative regulatory mechanisms of virus-mediated transcription of type I IFN include small ubiquitin-related modifier (SUMO) conjugation of both IRF3 and IRF7 as well as inhibition of IRF7-mediated transcription by ATF4, a key transactivator of the integrated stress responses [30], [31], [32]. In addition, rapid degradation of IRF3 was recently shown to play a critical role in the negative regulation of IFN-B gene expression during acute viral infection [33]. We have recently shown that IRF3 acts as a positive regulator of IFN-A genes by cooperating with IRF7 when low amounts of both factors are activated, but also can act as a repressor of IRF7-mediated transcription when the amount of IRF3 exceeds the amount of IRF7 [34]. Both factors exert their transcriptional effect by interacting with CBP/p300 and other HAT activities that target chromatin-associated histones during viral infection [35], [36], [37]. These data suggest that IRF3 can also participate in the negative regulation of IFN-A genes by preventing the recruitment of IRF7-associated HAT activities to IFN-A gene promoters or promoting the recruitment of histone deacetylase (HDAC) activities. The role of HDACs in regulating IFN-A gene expression has not been addressed. HDACs exert their repressive effect directly by removing the acetyl groups from amino termini of core histones, thereby generating a tight chromatin structure refractory to transcription, or indirectly by inhibiting HAT activities [38], [39]. Some HDACs associate with constitutively expressed co-repressors involved in basal repression of many inflammatory primary response genes, including cytokine genes, while their association with inducible repressors blocks the expression of secondary response genes, as part of a negative feedback mechanism that limits the inflammatory response [40], [41].

In the present study, we analyze the patterns of histone H3 acetylation at lysine residues K9 and K14, as well as the recruitment of HAT activities and HDAC3 to IFN-A gene promoters during virus infection. We demonstrate transient recruitment of HDAC3 to IFN-A gene promoters is in correlation with the post-inductional inhibition of IFN-A gene expression. Analysis of the recruitment patterns of IRF3 and IRF7 further shows that promoter occupancy by these factors modulates the recruitment of HDAC3. These data suggest that HDAC3 is required for inhibition of H3K9/K14 acetylation and attenuation of IFN-A gene transcription following Sendai virus infection.

## Materials and Methods

### Cell Culture, Virus Infection and Treatment of Cells with Actinomycin D

Human Namalwa cells (B lymphocytes from Burkitt’s lymphoma, ATCC CRL-1432), HEK293T cells (ATCC CRL-11268) and HEK293 cells constitutively expressing TLR3 (Invivogen, San Diego, CA) were grown in RPMI 1640, MEM and DMEM media (Invitrogen, Carlsbad, CA), respectively, supplemented with 10% fetal bovine serum (FBS), glutamine and antibiotics. Infection of cells with Sendai virus (SeV) was performed at a multiplicity of infection 1 to 2 (400–800 hemagglutinin units/2×10^6^ cells) in serum free media. After 1 h, cells were washed and placed in growth media containing 2% FBS, then harvested at different times following infection. For determination of IFN-A mRNA stability, actinomycin D (10 µg/ml, A1410, Sigma-Aldrich) was added directly to cell cultures following SeV infection for 8 h, and cells were collected at different time points after the addition of the inhibitor of transcription.

### Plasmids and Transient Transfections

The pcDNA3 series of plasmids expressing human IRF3, IRF7A, IKKε and various HDACs have been previously described [42], [43], [44], [45]. The pUNO-hTRAF3 plasmid encoding human TRAF3 was purchased from Invivogen. To test the effect of HDAC overexpression on virus-induced expression of IFN-A genes, HEK293-TLR3 cells (2×10^5^ cells grown in 6-well plates) were transfected with 0.60 or 1.50 µg of HDAC-encoding plasmid together with 0.15 µg of pcDNA3-IRF7A by lipofectamine 2000 (Invitrogen). An aliquot of total RNA extract from each sample was submitted to RT-QPCR analysis. HDAC expression was confirmed by anti-flag immunoblotting of the cell lysates.

### Small Interfering RNA Experiments

HEK293-TLR3 cells (2×10^5^ cells/6-well plates) were transfected with 80–160 pmols of target-specific siRNA for HDAC3 (sc-35538, Santa Cruz Biotech. CA) or scrambled siRNA (sc-37007) in the presence of 0.15 µg of pcDNA3-IRF7A and 0.45 µg of pUNO-TRAF3 and incubated for 40 h before infection by Sendai virus. Specificity and efficiency of siRNA on endogenous protein expression were tested by Western blotting, using whole cell extracts (20 µg of proteins separated on 10% SDS-polyacrylamide gel electrophoresis), HDAC3 polyclonal antibodies and α-actin antibodies used as control (sc-11417X and mAb-1501, respectively).

### Real-time RT-QPCR Analysis

An aliquot of total RNA (2 µg) was treated with DNase I and submitted to reverse transcription. QPCR analyses were performed as previously described [34]. QPCR assays were performed at least in triplicates using the ABsolute™ QPCR SYBR® Green ROX (500nM) mix (ABgene, Epsom, UK) in 7900HT Fast Real-Time PCR System apparatus (Applied Biosystem, Foster City, CA). Primer pairs used to determine the expression levels of IFN-A, IFN-B, IRF3, IRF7, HDACs, and GAPDH genes have been previously described [34], [46]. The amplification product was identified by DNA sequencing and checked by the analysis of the melting curve in each assay. Relative expression levels are presented as mRNA copies per 100 GAPDH mRNA copies. Standard deviation values indicated in the histograms derive from at least two independent experiments performed in triplicate.

### Chromatin Immunoprecipitation (ChIP) Assays

Sendai virus-infected Namalwa B cells (5×10^6^ cells/100 mm diameter plate) and HEK293T-TLR3 cells (2×10^6^ cells/100 mm plate) transfected with 0.75 µg of pcDNA3-IKKε and 1.5 µg of pcDNA3-IRF3wt, pcDNA3-IRF7A or both IRF-expressing plasmids were analyzed by ChIP assays, as previously described [34]. Briefly, cross-linked chromatin (3 to 5 µg) purified by isopycnic centrifugation was incubated overnight at 4°C with 1.2 to 2 µg of specific antibodies listed below and with rabbit IgG sera (goat IgG sera for PCAF and GCN5) used as control. DNA recovered after immunoprecipitation was used as template for QPCR with different primer sets amplifying the IFN-A promoter fragments that include all the binding sites for IRF3 and IRF7. Forward primers GGAACAAGATGGGGAAGACA, AAGGCTCT GGGGTAAAAGA, TTTGAGTGCAGGGGAAA AAC and AAGCCCATGGGGCAGGGAA, and reverse primers GCAGATACTTCTGGGCTTGC, GACCTTGCTTTGTGCCTAGC, CGTGGCCTCT AGGTTTTCTG and GGGCTGGTTGATGA GGGGT were used for IFN-A1 [−178 to +59], A2 [−184 to +24], A7 [−177 to +39] and A14 [−184 to +49] promoters, respectively. The specificity of amplification was confirmed by DNA sequencing. Standard curves obtained with 4-log range dilutions of genomic DNA extracted from HEK293T cells were used to determine the amplification efficiency of each primer set (1.85 for IFN-A1, 1.73 for IFN-A2, 1.99 for IFN-A7, and 1.66 for IFN-A14). Primers designed to amplify the c-fos gene promoter (Diagenode, Liège, Belgium) were used as control. Primers used to determine the expression levels of IFN-A1, A2, A7 and A14 genes were also used to amplify the coding region of these genes in ChIP experiments.

### Immunoblot Analyses

Whole cell extracts were prepared from Namalwa B cells infected by Sendai virus. Proteins (20 µg) were separated on 10% SDS-PAGE and analyzed by Western blotting using anti-HDAC3 antibodies or α-actin.

### Antibodies

Anti-acetylated histone H3-K9 and H3-K14 antibodies (07-352 and 07-353, Millipore) generated for use in ChIP analysis are highly specific for these acetylation sites; their specificity was also confirmed by peptide arrays [47], [48]. Antibodies for IRF3, IRF7, GCN5, PCAF, CBP, p300, HDAC1, HDAC3 and histone H3 (sc-9082X and sc-9083X, sc-583X, sc-584X, sc-6303X, sc-6300X, sc-7872X, sc-11417X and sc-10899X, Santa Cruz, respectively) used in ChIP experiments have been previously described [34], [49], [50], [51], [52]. TBP antibodies were obtained from Diagenode (Liège, Belgium). HDAC3 antibodies and α-actin (mAb-1501, Millipore Co. Ma, USA) were used in Western blot analysis.

### Data Analysis and Statistics

Two-tailed paired Student’s t-tests were used for generating *p*-values to determine statistical significance of virus-induced gene expression, siRNA knockdown and ChIP-QPCR experiments. In all analyses, the threshold for statistical significance was *p*<0.01. In ChIP experiments, the relative proportions of co-immunoprecipitated promoter fragments were determined based on the threshold cycle (T_C_) value obtained by QPCR, after reversion of the cross-link. Values obtained with input chromatin DNA (T_C input_) and values obtained with chromatin precipitated in the presence of specific antibody (T_C target_) were determined in duplicate from at least two independent immunoprecipitations (n = 4) for each IFN-A gene promoter. Histone H3 modifications associated to the IFN-A gene promoter and recruitment of TBP, IRFs and HDACs were determined by raising the amplification efficiency to the power of (T_C input_ - T_C target_). The results obtained at different time points were expressed as hundredths (%) of the DNA input. T_C IgG_ values obtained with chromatin incubated with IgG sera were used to determine unspecific binding. Enrichment fold values were calculated by raising the amplification efficiency to the power of (T_C IgG_ − T_C target_), and used to determine statistical significance. Mean ± SD values of enrichment folds are presented.

## Results

### Transcriptional Inhibition of Virus-induced IFN-A Gene Expression

**Figure 1:**
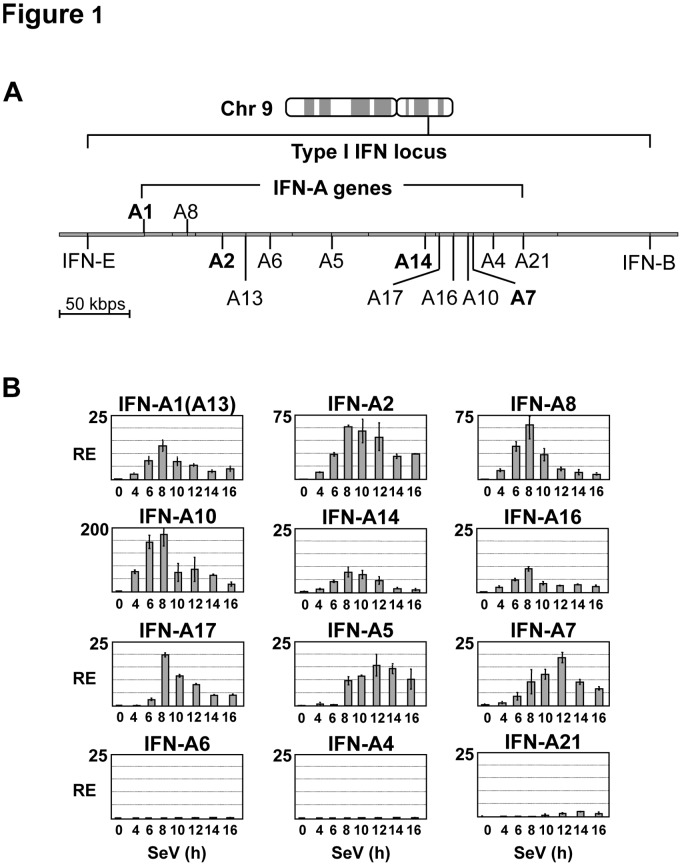
Expression pattern of IFN-A genes in Namalwa B cells infected by Sendai virus. (**A**) Type I IFN locus, delimited to 400 kbps on human chromosome 9, consists of thirteen intronless IFN-A genes located in cluster with the IFN-B and IFN-E genes (data retrieved from NCBI library, version 37.1). IFN-A genes analyzed in this study are indicated in bold in the diagram. IFN-A1 and IFN-A8 are in reverse orientation compared to other IFN-A genes. (**B**) IFN-A gene expression was determined by quantitative RT-QPCR analysis in Namalwa B cells mock-infected (0 h) or infected by Sendai virus for 4–16 h. IFN-A1 expression levels presented in this study represent the total amounts of both IFN-A1 and IFN-A13 mRNAs; the high percentage of sequence homology of IFN-A1 and IFN-A13 genes (99.7% in the coding region) did not allow distinction between these genes. Relative expression (RE) normalized to GAPDH mRNA levels is indicated with standard deviation values calculated in two independent experiments performed in duplicate.

In the present study, Namalwa B lymphoid cells infected with Sendai virus (SeV) were used as a model to determine the kinetics of IFN-A gene activation, with each functional IFN-A gene present in the type I IFN locus monitored by RT-QPCR. IFN-A genes exhibited transient expression with mRNA levels accumulating between 4 and 8 h (IFN-A1, A2, A8, A10, A14, A16 and A17) or between 6 and 12 h (IFN-A5 and A7) post-infection (p.i), whereas IFN-A4, A6 and A21 genes were weakly induced (**Fig. 1A-B**). IFN-A gene expression decreased after 8 h and 12 h of infection, for the first and second groups, respectively. Decrease in mRNA levels was independent of actinomycin D addition, as shown for IFN-A1, A2 and A14 (**Fig. S1**), suggesting that attenuation of IFN-A gene expression was due to transcriptional inhibition.

**Figure 2:**
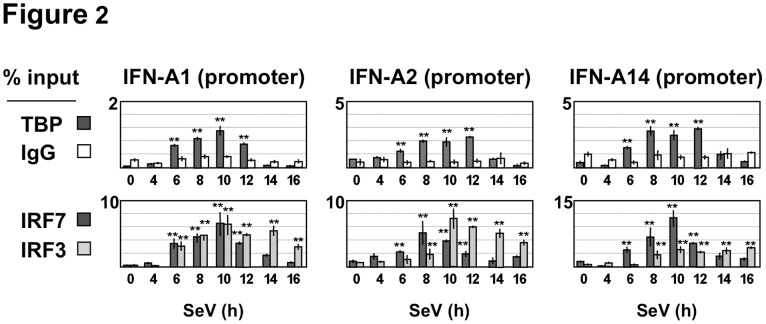
Recruitment of IRF3, IRF7 and TBP to IFN-A gene promoters. Recruitment to the IFN-A2, A14 and A1 gene promoters was determined by ChIP-QPCR assays in Namalwa B cells infected by Sendai virus. Cross-linked chromatin extracts were immunoprecipitated with antibodies specific for TBP, IRF7 or IRF3 and submitted to QPCR analysis after reversion of cross-linking, as described in Materials and Methods. Anti-rabbit IgG was used to determine background levels of non-specific binding. Data are expressed as % of the DNA input. Values are mean and standard deviation for two replicate samples derive from two representative experiments (n = 4). The error bars indicate the relative standard deviations.

ChIP-QPCR experiments were next performed to establish whether transient IFN-A gene expression correlated with the recruitment pattern of TATA-binding protein (TBP) to different IFN-A gene promoters following virus infection. The high sequence homology within IFN-A gene promoter regions, extending 400 bp upstream from the transcription initiation site [53], [54], prompted us to select IFN-A1, A2, A7 and A14 genes for analysis. Recruitment of TBP to the IFN-A promoters was detected at 6–12 h p.i (an average of 2% of the input, with p<0.01), with 2- to 4-fold enrichment calculated for IFN-A2, A14 and A1 (**Fig. 2**). TBP recruitment correlated with maximal IFN-A gene expression, and decreased at 14 h p.i (% of the input values comparable to those determined in uninduced cells). These results indicated that the removal of TBP from IFN-A gene promoters was concomitant with the inhibition of gene expression, occurring rapidly after gene induction. We also determined the dynamics of IRF3 and IRF7 recruitment to the IFN-A gene promoters in the course of virus infection. Both factors were recruited to the IFN-A1, A2 and IFN-A14 gene promoters following virus infection (**Fig. 2**); occupancy by IRF7 and IRF3 reached maximal values at 8–10 h; IRF7 enrichment of the promoters then decreased at 14 h p.i, concomitant with transcriptional inhibition, while significant recruitment of IRF3 was observed up to 16 h following infection. These data indicated that virus-induced IFN-A gene expression correlated with the transient recruitment of IRF7 and TBP to the IFN-A gene promoters. They also indicated that removal of both IRF7 and TBP from the IFN-A gene promoters was associated with considerably reduced levels of IFN-A gene expression. Higher levels of IRF7 recruitment to IFN-A14 gene promoter (in comparison to IFN-A1 or A2) were in agreement with previous results indicating the high responsiveness of this promoter to IRF7-mediated transcription [22]. Similarly, high levels of IRF3 recruitment might be related to the presence of IRF sites exhibiting preferential binding for IRF3 in the IFN-A1 and A2 gene promoters [22]. Sustained recruitment of IRF3 from 8 to 16 h p.i to IFN-A gene promoters during virus-induced activation and inhibition of gene expression suggested that this factor might be involved both in the initiation and post-inductional repression of IFN-A gene transcription.

### Transient Histone H3K9 Acetylation Associated with IFN-A Gene Promoters

**Figure 3:**
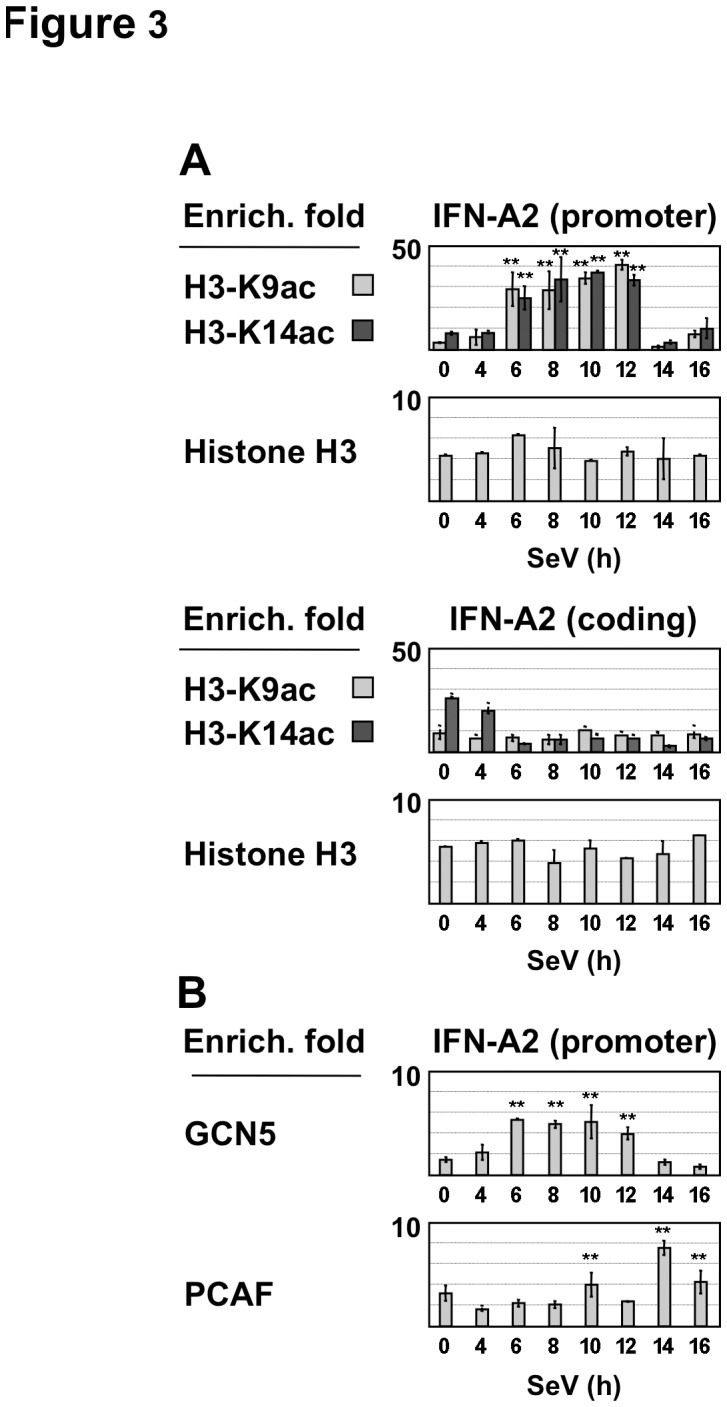
Histone H3K9/K14 acetylation pattern associated with the IFN-A gene promoters during virus infection. (**A**) Acetylated histone H3K9, acetylated H3K14 and histone H3 associated with the promoter and the coding region of the IFN-A2 gene were determined by quantitative ChIP assays in Namalwa B cells infected by Sendai virus. Mean and standard deviation values of enrichment fold was calculated as described in Materials and Methods, with *p* values of statistical significance lesser than 0.01 indicated by **. These values derived from two replicate samples in two representative experiments. Anti-acetylated histone H3K9 and anti-acetylated H3K14 antibodies used in these experiments have shown to be highly specific for these acetylation sites [47], [48]. Anti-rabbit IgG serum was used as control of ChIP experiments. (**B**) Enrichment folds for the recruitment of GCN5 and PCAF to the IFN-A2 promoter were determined as described in Figure 2. Anti-goat IgG serum was used as control of ChIP experiments.

Genome-wide analyses of histone acetylation have demonstrated that the acetylation of individual lysines in histone H3 and H4 tails and more specifically histone H3 acetylation at lysines K9 and K14 correlates with the active state of gene transcription [55], [56], [57]. To further understand the involvement of histone acetylation in regulating IFN-A gene expression, the modulation of histone H3K9 and H3K14 acetylation at the IFN-A promoters was examined in SeV-infected Namalwa B cells. At 6–12 h p.i, robust H3K9 and H3K14 acetylation was detected, with H3K9 acetylation levels 3- to 5-fold higher than those detected earlier (4 h) or later (14–16 h) times after infection (**Fig. 3A** **and Fig. S2A**). Virus-infection increased H3K14 acetylation by 2- to 3-fold compare to the constitutive levels detected in uninfected cells. ChIP experiments with histone H3 antibodies indicated that nucleosome occupancy remained stable in the promoter region (**Fig. 3A**), thus confirming that promoter-specific histone H3K9 and H3K14 acetylation was modulated during virus infection. Accordingly, histone acetylation of the IFN-A promoters was barely detectable in uninfected Namalwa B cells at different time points (**Fig. S3A**). Low levels of histone H3 acetylation were observed within the coding region of IFN-A genes, suggesting that virus-infection induced local acetylation at the IFN-A gene promoter regions (**Fig. 3A**). As a negative control, virus infection did not induce c-fos gene expression and did not modify histone H3 marks associated with the c-fos promoter (**data not shown**). Negative controls also included PCR amplifications performed with the chromatin extracts in the absence of antibodies or with chromatin precipitated in the presence of irrelevant antisera (**Fig. S3B**). H3K9 and H3K14 acetylation associated with IFN-A gene promoters strongly decreased at 14–16 h p.i (**Fig. 3A** and **Fig. S2A**). Furthermore, inhibition of H3K9 and H3K14 acetylation correlated with removal of IRF7 and TBP from the IFN-A promoters and inhibition of transcription (**see** **Figs. 1** **and** **2**). These results indicated that inhibition of SeV-induced IFN-A gene transcription was related to inhibition of histone H3K9/K14 acetylation in the IFN-A gene promoters.

To identify the histone acetyltransferase activities (HAT) responsible for acetylation of H3K9 and H3K14 [58], [59], we analyzed the binding of PCAF and GCN5 that have been shown to contribute to sequential H3K9 and K14 acetylation of the IFN-B gene promoter during SeV infection [60], [61]. Low levels of GCN5 recruitment to the IFN-A gene promoters (enrichment by 3- to 5-fold with IFN-A2, and by 2-fold with IFN-A1 and A14) were detected at 6–12 h p.i, while PCAF recruitment was observed at 10 h and 14–16 h p.i (**Fig. 3B** **and Fig. S2B**). Thus, transient recruitment of GCN5, but not that of PCAF, correlated well with H3K9 and K14 acetylation, suggesting that GCN5 might be responsible for virus-induced histone H3 acetylation of the IFN-A promoters.

### The Inhibitory Effect of HDAC3 on Virus-induced IFN-A Gene Transcription

**Figure 4:**
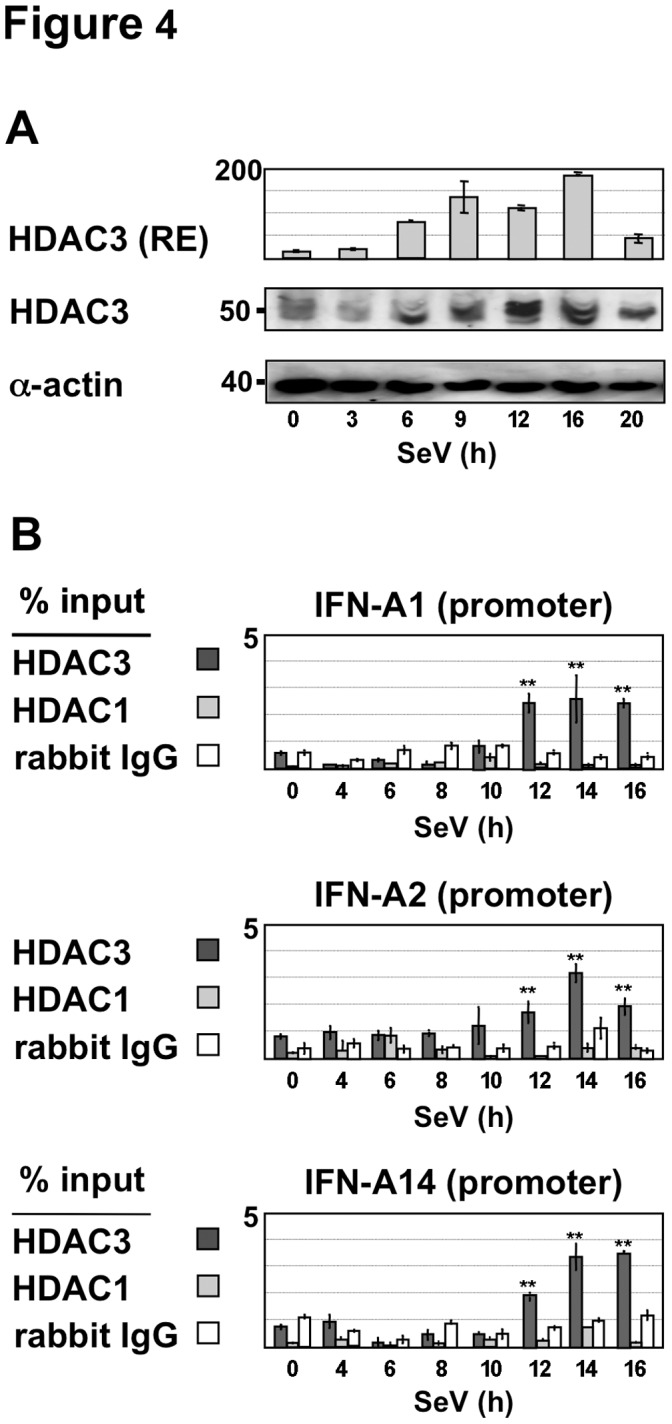
Recruitment of HDAC3 to IFN-A gene promoters during virus infection. (**A**) Relative expression levels of HDAC3 were determined in Sendai virus-infected Namalwa cells by RT-QPCR as described in Figure 1. HDAC3 protein levels were analyzed in whole cell extracts prepared from Sendai virus-infected Namalwa B cells by Western blotting using HDAC3 and actin antisera. (**B**) Recruitment of HDAC3 and HDAC1 to the IFN-A2, A14 and A1 gene promoters was determined in Namalwa B cells infected by Sendai virus for 4 to 16 h by quantitative ChIP assays as described in Figure 2. Anti-rabbit IgG was used to determine non-specific binding.

Several studies have implicated HDAC activities in reversal of histone acetylation and attenuation of a transcriptional response [62], [63]. We observed that infection of Namalwa cells by SeV resulted in a strong and transient increase in HDAC3 mRNA and protein levels at 9–16 h p.i (**Fig. 4A**). In Western blots, in addition to the 50-kDa form of HDAC3, we detected a slower migrating complex reacting with HDAC3 antibodies that might correspond to a post-translationally modified form of HDAC3 that appears during virus infection. Because virus infection stimulated HDAC3 expression, we tested whether HDAC3 was recruited to IFN-A gene promoters during virus infection. Interestingly, HDAC3 recruitment to different IFN-A gene promoters was observed at 12–16 h p.i, with levels ranging from 2 to 3% of the input DNA and providing a 3- to 6-fold enrichment (*p*<0.01), whereas other HDACs including HDAC1 were not recruited (**Fig. 4B** **and data not shown**). These results indicated that the inhibition of H3K9/K14 acetylation at 14–16 h p.i correlated with HDAC3 recruitment to IFN-A promoters.

**Figure 5:**
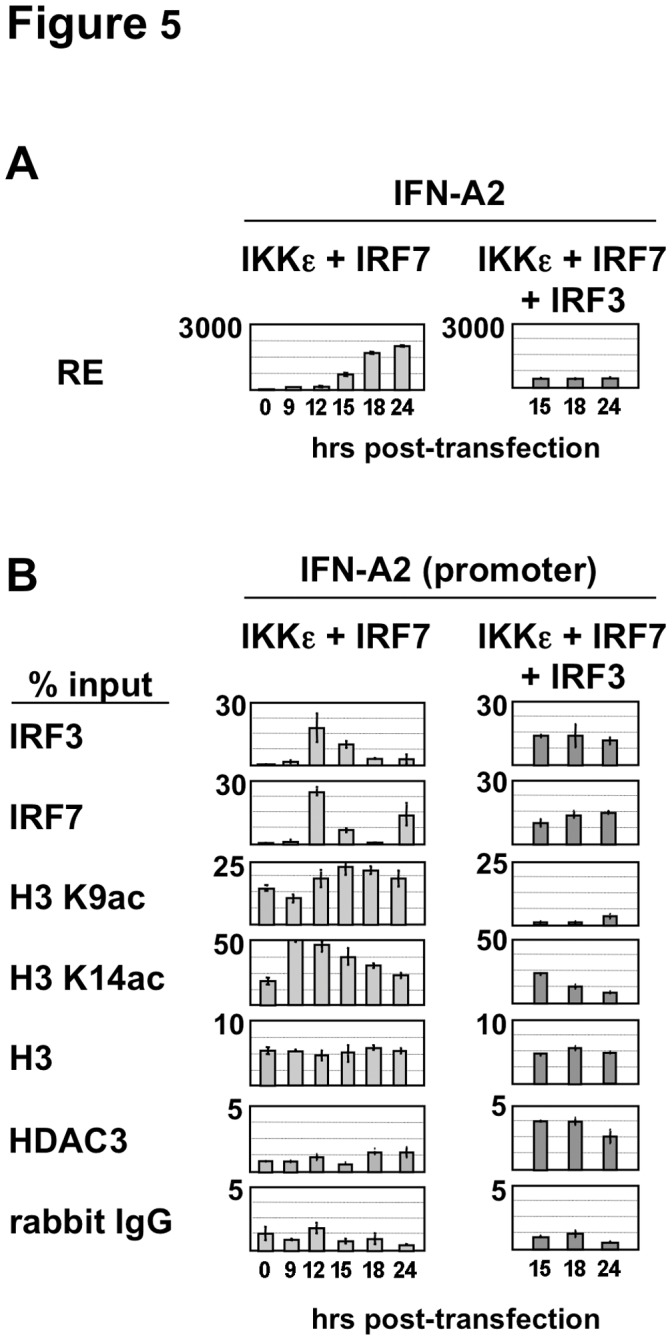
Regulation of histone H3 acetylation associated with the IFN-A2 gene promoter. (**A**) IFN-A2 expression was determined in HEK293-TLR3 cells co-expressing IKKε and IRF7 alone or together with IRF3. Cells were collected for RT-QPCR analysis at the indicated times (in hours) after transfection. (**B**) Acetylated histone H3K9, acetylated H3K14 and histone H3, as well as recruitment of IRF3, IRF7 and HDAC3 associated with the IFN-A2 gene promoter were determined by quantitative ChIP assays as described in Materials and Methods. Anti-rabbit IgG was used to determine non-specific binding.

To investigate further the involvement of HDAC3 in inhibition of IFN-A transcription, via H3K9 and H3K14 deacetylation, transient expression studies were performed in HEK293-TLR3 cells co-expressing IRF7 in the presence of IKKε. In this model, active IFN-A transcription correlated with recruitment of IRF7 and histone H3 acetylation, whereas no HDAC3 recruitment was observed (**Fig. 5A-B**). We have shown that IRF3 cooperates with IRF7 when low amounts of both factors are expressed, but also can act as a repressor of IRF7-mediated transcription when expressed at higher amounts compared to IRF7 [34]. Interestingly, when high levels of both IRF3 and IRF7 were activated in the presence of IKKε, we observed significant levels of HDAC3 recruitment (2.5 to 3.7% of input, with p<0.01) that correlated with a decrease in H3K9 and H3K14 acetylation and an impaired IFN-A2 gene expression (**Fig. 5A-B**). In this experiment, a 5- to 20-fold reduction in H3K9 acetylation and 2-fold reduction in H3K14 acetylation was observed compared to H3 acetylation levels in cells expressing IKKε and IRF7.

**Figure 6:**
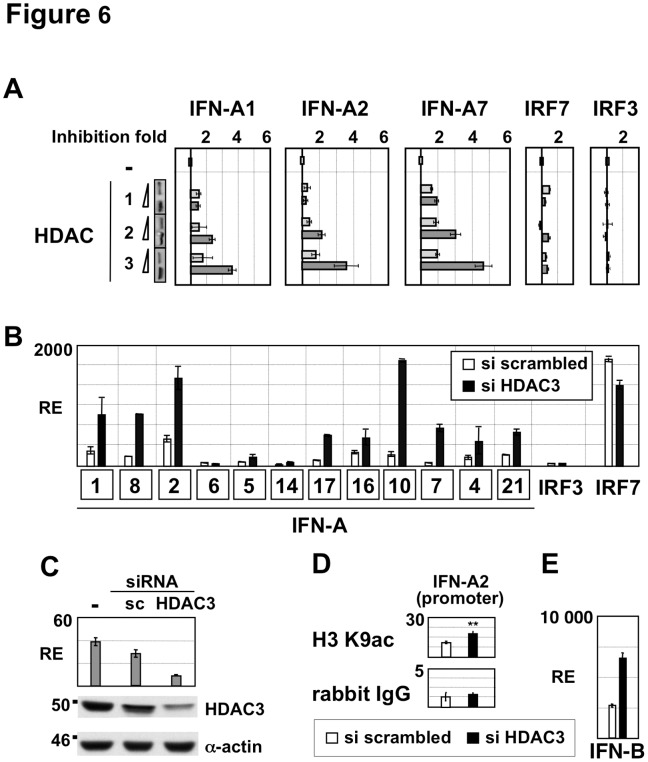
Inhibitory effect of HDAC3 on IFN-A gene transcription. (**A**) The effect of HDACs class I (1, 2 and 3) on IFN-A gene transcription was determined in HEK293-TLR3 cells transfected with pcDNA3-IRF7A together with an HDAC-encoding plasmid added in 2-fold increasing amounts, as indicated in Materials and Methods. After 24 h of expression, the inhibition fold was calculated from IFN-A mRNA levels determined by RT-QPCR in HDAC-expressing cells in comparison to control cells transfected with pcDNA3. HDAC expression determined by anti-flag immunoblotting of the cell lysates is shown in the insets. (**B**) HDAC3 expression was specifically inhibited for 40 h by siRNA in HEK293-TLR3 cells expressing TRAF3 and IRF7A. IFN-A gene expression was determined after HDAC3 depletion by RT-QPCR following 8 h of infection by Sendai virus and compared to the expression determined in cells transfected with scrambled siRNA oligonucleotides. (**C**) Silencing efficiency was confirmed by RT-QPCR and Western blot analyses using total RNA and proteins extracted from cells transfected with siRNA oligonucleotides specific for HDAC3 or a mixture of scrambled (sc) siRNAs. (**D**) Histone H3K9 acetylation associated with the IFN-A2 gene promoter was determined in control and HDAC3-depleted cells by quantitative ChIP assays as described in Materials and Methods. Anti-rabbit IgG was used to determine non-specific binding. (**E**) IFN-B gene expression was determined by RT-QPCR following 8 h of infection by Sendai virus in HEK293-TLR3 cells expressing TRAF3 and IRF7A and transfected with scrambled siRNA or siRNA oligonucleotides specific for HDAC3.

In the same experimental model, HDAC3 overexpression produced a 2–4 fold dose-dependent inhibition of IRF7-mediated IFN-A gene expression without altering expression of either IRF3 or IRF7 (**Fig. 6A**). Other HDACs tested did not exert similar effects (**Fig. S4**). Conversely to the inhibitory effect of HDAC3 overexpression, silencing of HDAC3 expression by siRNA led to a 2-3-fold decrease in HDAC3 mRNA and protein levels and enhanced virus-induced IFN-A gene expression by 3- to 5-fold compared to cells transfected with control siRNA (**Fig. 6B-C**). Also, a significant 1.5 fold increase (p<0.01) in histone H3K9 acetylation of the IFN-A2 promoter was quantified in cells transfected with siRNA targeting HDAC3 (**Fig. 6D**). Since a knockdown screen performed in poly(I).poly(C)-treated cells showed that HDACs affected negatively (HDAC1 and HDAC8) or positively (HDAC6) the activity of a IFN-B promoter construct in reporter assays [64], we tested the effect of HDAC3 knockdown on SeV-induced IFN-B expression. Depletion of HDAC3 slightly enhanced SeV-induced IFN-B gene transcription by less than 2.5 fold (**Fig. 6E**) and overexpression of HDAC3 did not affect virus-induced IFN-B gene transcription (data not shown). These observations suggested that HDAC3 did not play a critical role in IFN-B gene regulation following virus infection.

Taken together, these results indicated that recruitment of HDAC3 to IFN-A gene promoters led to histone H3K9 (and H3K14) deacetylation and post-inductional inhibition of IFN-A gene transcription.

## Discussion

In the present study, we demonstrate an important role for histone H3 acetylation in the stimulation of IFN-A gene transcription during virus infection, and a functional involvement of HDAC3 in the post-induction repression of IFN-A gene transcription, acting *via* histone H3K9 and H3K14 deacetylation.

During Sendai virus infection of Namalwa cells, many of the genes of the IFN-A locus - IFN-A1, A2, A5, A7, A8, A10, A14, A16, and A17 - are actively transcribed, reaching a peak of mRNA expression at 8–12 h after infection. It has been reported that Sendai virus triggers type I IFN gene transcription by specific signaling pathways that culminate in the activation of both IRF3 and IRF7 [2], [65], [66]. Here, we show that peak levels of transcription correspond to transient recruitment of IRF7 and IRF3 to the promoters during virus infection. At early times (6–12 h after virus infection), promoter occupancy by IRF3 and IRF7 is associated with transient recruitment of GCN5 and virus-induced histone H3K9 and H3K14 acetylation of IFN-A gene promoters. Beginning at approximately 14 h after infection, decreased levels of IFN-A mRNA correlated with decreased histone H3K9 and H3K14 acetylation and recruitment of HDAC3 to IFN-A gene promoters. IFN-A genes (IFN-A1, A2, A7 and A14) selected for detailed analysis are spaced by 55, 145 and 38 kbps, respectively, from one another in the type I IFN locus of 400 kbps [53], [67]. Ordered recruitment of GCN5 and HDAC3 activities to these promoters, starting at 6 h and 12 h, respectively, clearly indicated that the IFN-A gene transcription was coordinately regulated during virus infection. The involvement of HDAC3 in negative regulation of IFN-A gene transcription is based on the observations that: i) silencing of HDAC3 expression by siRNA enhanced both virus-induced IFN-A gene transcription and histone H3K9 acetylation of the IFN-A gene promoters; and ii) HDAC3 overexpression induced a dose-dependent inhibition of IRF7-mediated IFN-A gene expression. Although the role of other HDACs in IFN-A gene regulation may not be excluded, class I and class II HDACs did not display significant inhibitory effect on IRF7-mediated transcription. We therefore focused our analysis on the effect of HDAC3 on IFN-A gene regulation.

Several studies have correlated IFN-A gene transcription with the recruitment of IRF3 and/or IRF7 to IFN-A promoters [5], [54], [68], [69]. Other reports have demonstrated that cooperative binding of ATF2/c-Jun, IRF3, IRF7, and NF-κB to the IFN-B promoter induces the ordered recruitment of GCN5, PCAF, chromatin-remodeling SWI/SNF complex, CBP-Pol II holoenzyme and TFIID to induce histone H3K9 and H3K14 acetylation and virus-induced IFN-B gene expression [14], [15], [60], [70]. HDAC3 involvement in the IFN-A gene regulation was further supported by the observations that activation of both IRF7 and IRF3 led to sustained HDAC3 recruitment, impaired histone H3K9 acetylation and attenuated IFN-A gene transcription, whereas activation of IRF7 led to robust histone H3K9/K14 acetylation and high levels of IFN-A gene expression, without HDAC3 recruitment. These results suggest that promoter occupancy by IRF3 and IRF7 differentially regulates the recruitment of both GCN5 and HDAC3 to sequentially activate and repress IFN-A gene transcription during virus infection. Particular nucleotide substitutions occurring in the virus-responsive element (VRE) of IFN-A gene promoters severely affect the selective binding of IRF3 or IRF7, while others impair the binding of both factors, thereby determining differential virus-inducible expression levels of IFN-A genes [34], [42], [68], [69]. These substitutions might account for the differences observed in the expression kinetics of IFN-A genes analyzed in this study.

The mechanism of HAT and HDAC3 recruitment to IFN-A gene promoters remains to be determined. Reversal of induced histone hyperacetylation by HDACs following the removal of an inducing signal has been reported [62], [63], [71], [72]. Specifically, HDAC3 negatively regulates the transcription of inflammatory response genes by directly interacting with co-repressors recruited to target gene promoters by various transcription factors [41], [73], [74], [75]. As both IRF3 and IRF7 exert their transcriptional effect by interacting with CBP/p300 and other HAT activities [35], [36], [37], these coactivators might participate to the recruitment of HDAC activities that target chromatin-associated histones during viral infection. Association of HDAC3 with p300 has been involved in chromatin deacetylation and repression of the c-Myc promoter transcription [76]. IRF3 might also participate, through its association with p300, to the recruitment of HDAC3 to the IFN-A gene promoters. We detected substantial amounts of IRF3 associated with the IFN-A gene promoters during inhibition of virus-induced H3K9 and H3K14 acetylation. During this inhibitory period, H3K14 hyperacetylation of IFN-A gene promoters was less reduced in comparison to the decrease observed in H3K9 acetylation. Relative maintenance of H3K14 acetylation might be due to HAT activities (CBP or p300) associated with IRF3 [58], [77].

In summary, our present results support the following model of IFN-A gene regulation: promoter occupancy by IRF3 and IRF7 at early times after SeV infection (6–12 h) stimulates transcriptional induction of the IFN-A gene locus and is associated with chromatin modification via histone acetylation involving H3K9 and H3K14 acetylation (and other post-translational histone modifications of chromatin). At later times (14–24 h) after infection, IRF7 and TBP are released from the promoter, concomitant with histone H3K9 and H3K14 deacetylation; the attenuation of IFN-A gene transcription. H3K9 deacetylation correlates directly with the recruitment of HDAC3 to IFN-A promoters, thus providing a mechanism to maintain controlled expression of IFN-A genes.

## Supporting Information

Figure S1 **Effect of Actinomycin D on the post-inductional inhibition of IFN-A gene expression.** RT-QPCR analysis of IFN-A1, A2, and A14 mRNA transcripts was performed in the presence (black squares) of actinomycin D (ActD) added after 8 h of infection or in the absence of ActD (open squares). Relative expression of IFN-A genes obtained in two independent experiments was normalized to GAPDH mRNA levels and plotted in binary logarithm as a function of time.
(TIF)

Figure S2 **Virus-induced histone H3 acetylation pattern associated to IFN-A gene promoters.** (**A**) H3K9 and H3K14 acetylation levels associated to IFN-A1, A7 and A14 gene promoters and to their coding regions were determined by ChIP-QPCR in Namalwa B cells infected by Sendai virus, as described in **Figure 3A**. (**B**) Recruitment of GCN5 and PCAF to the IFN-A1 and A14 promoters was determined by quantitative ChIP assays in Namalwa B cells infected by Sendai virus as described in **Figure 3B**.
(TIF)

Figure S3 **Negative controls of ChIP-QPCR experiments.** (**A**) Constitutive H3K9 and H3K14 acetylation levels associated to IFN-A1, A2, A7 and A14 gene promoters were determined by ChIP-QPCR in Namalwa B cells at different time points, as described in **Figure 3A**. (**B**) ChIP-QPCR experiments carried out with chromatin extracts in the absence of antibodies or in the presence of actin antibodies using primers for IFN-A1, A2, A7 and A14 gene promoters were performed in Namalwa B cells infected by Sendai virus, as described in **Figure 3A**.
(TIF)

Figure S4 **Effect of HDAC overexpression on IRF7-mediated IFN-A gene transcription.** The effect of HDACs class I (1, 2 and 3), class IIa (4, 5, and 7) and class IIb (6 and 10) on IFN-A and IRF gene transcription was determined in HEK293-TLR3 cells transfected with pcDNA3-IRF7A together with an HDAC-encoding plasmid added in 2-fold increasing amounts. After 24 h of expression, the inhibition fold was calculated from IFN-A mRNA levels determined by RT-QPCR in HDAC-expressing cells in comparison to control cells transfected with pcDNA3. Expression levels of each HDAC determined by anti-flag immunoblotting of the cell lysates are shown in the insets.
(TIF)
