# Supplementary figures and images for: Recruitment of Histone Deacetylase 3 to the Interferon-A Gene Promoters Attenuates Interferon Expression
Source: PLoS One. 2012 Jun 7;7(6):e38336. doi: 10.1371/journal.pone.0038336 (PMC3369917; doi:10.1371/journal.pone.0038336)

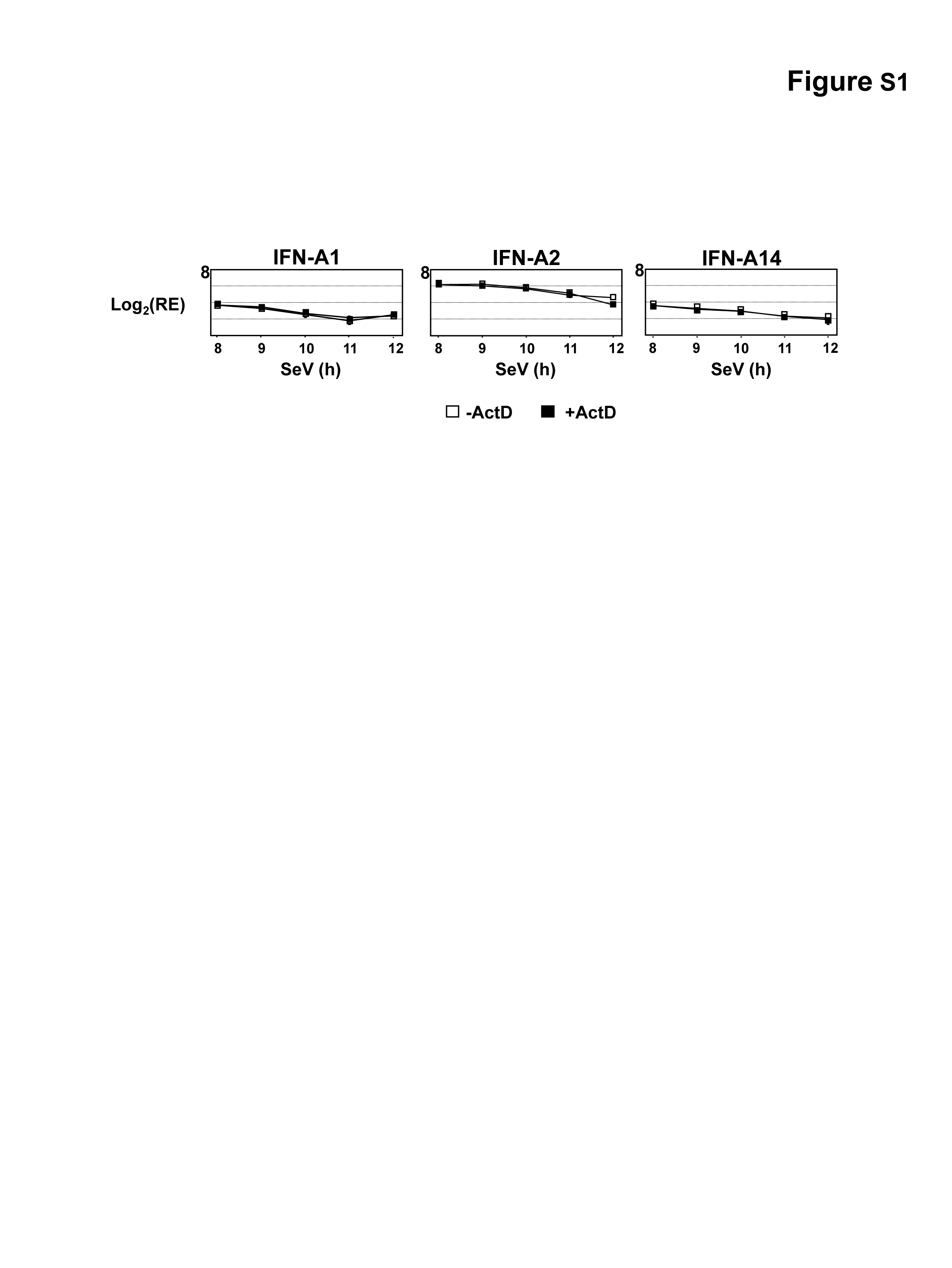

Supplement: Figure S1 — Effect of Actinomycin D on the post-inductional inhibition of IFN-A gene expression. RT-QPCR analysis of IFN-A1, A2, and A14 mRNA transcripts was performed in the presence (black squares) of actinomycin D (ActD) added after 8 h of infection or in the absence of ActD (open squares). Relative expression of IFN-A genes obtained in two independent experiments was normalized to GAPDH mRNA levels and plotted in binary logarithm as a function of time. (TIF) [file pone.0038336.s001.tif]

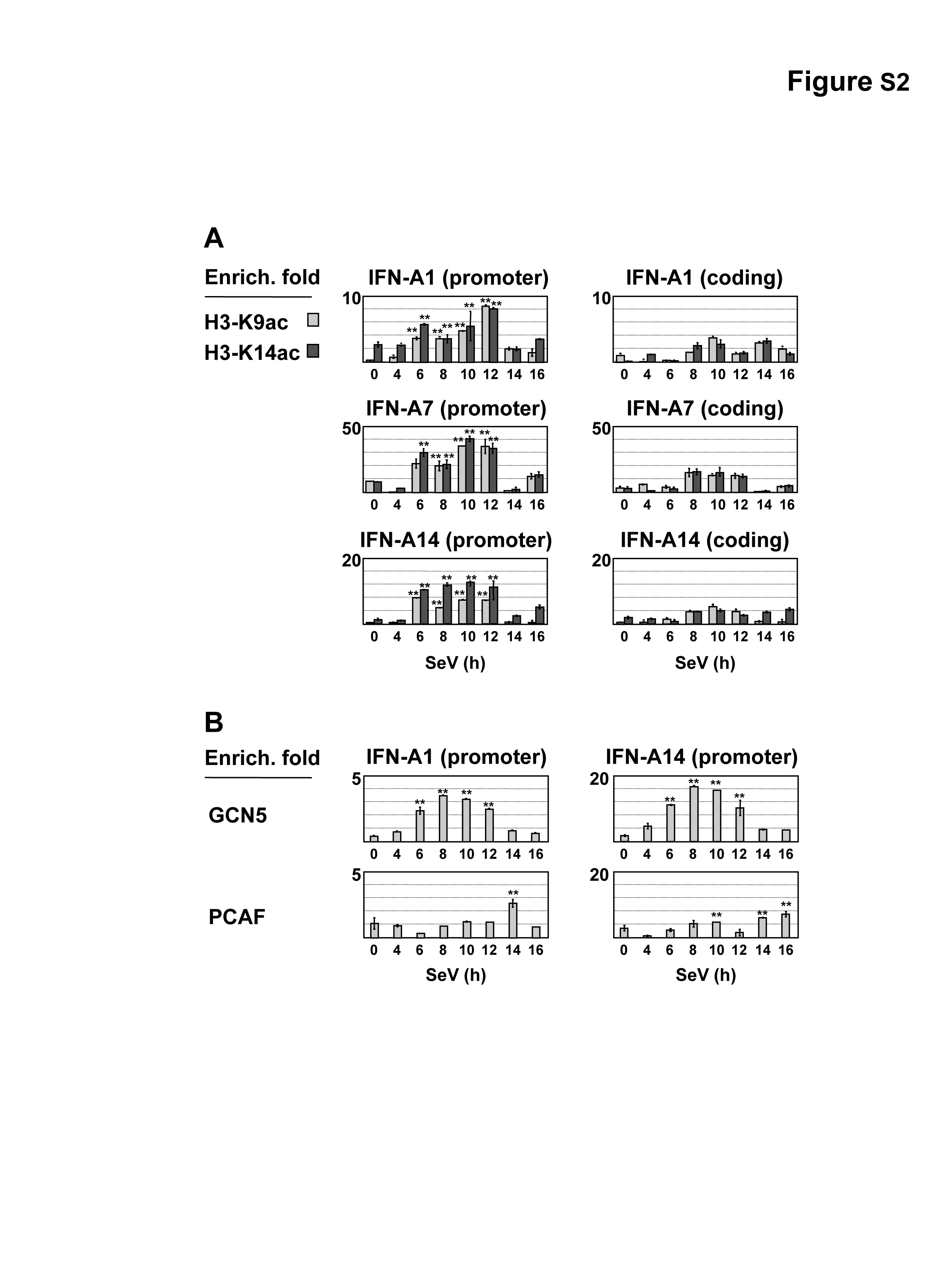

Supplement: Figure S2 — Virus-induced histone H3 acetylation pattern associated to IFN-A gene promoters. (A) H3K9 and H3K14 acetylation levels associated to IFN-A1, A7 and A14 gene promoters and to their coding regions were determined by ChIP-QPCR in Namalwa B cells infected by Sendai virus, as described in Figure 3A . (B) Recruitment of GCN5 and PCAF to the IFN-A1 and A14 promoters was determined by quantitative ChIP assays in Namalwa B cells infected by Sendai virus as described in Figure 3B . (TIF) [file pone.0038336.s002.tif]

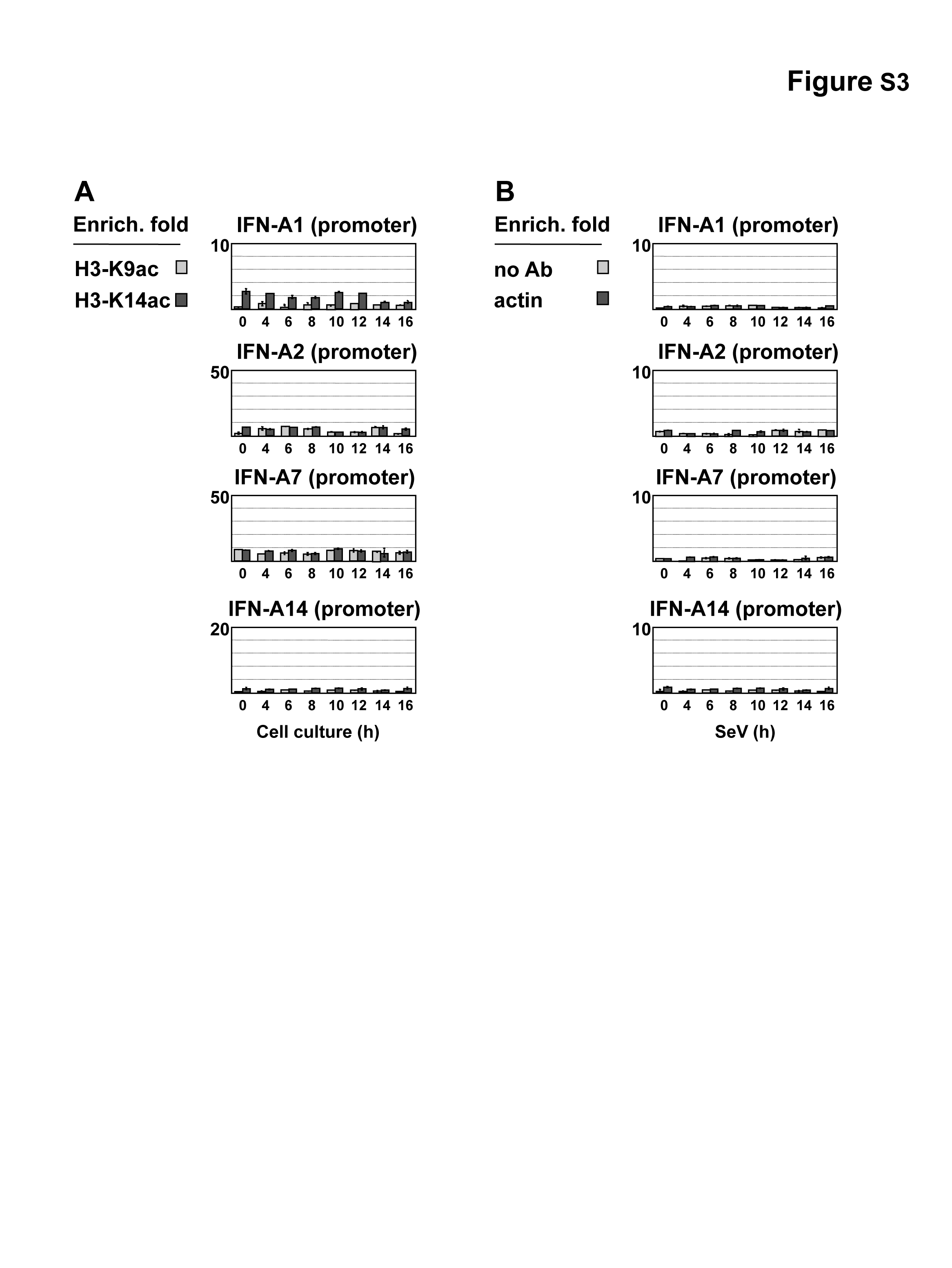

Supplement: Figure S3 — Negative controls of ChIP-QPCR experiments. (A) Constitutive H3K9 and H3K14 acetylation levels associated to IFN-A1, A2, A7 and A14 gene promoters were determined by ChIP-QPCR in Namalwa B cells at different time points, as described in Figure 3A . (B) ChIP-QPCR experiments carried out with chromatin extracts in the absence of antibodies or in the presence of actin antibodies using primers for IFN-A1, A2, A7 and A14 gene promoters were performed in Namalwa B cells infected by Sendai virus, as described in Figure 3A . (TIF) [file pone.0038336.s003.tif]

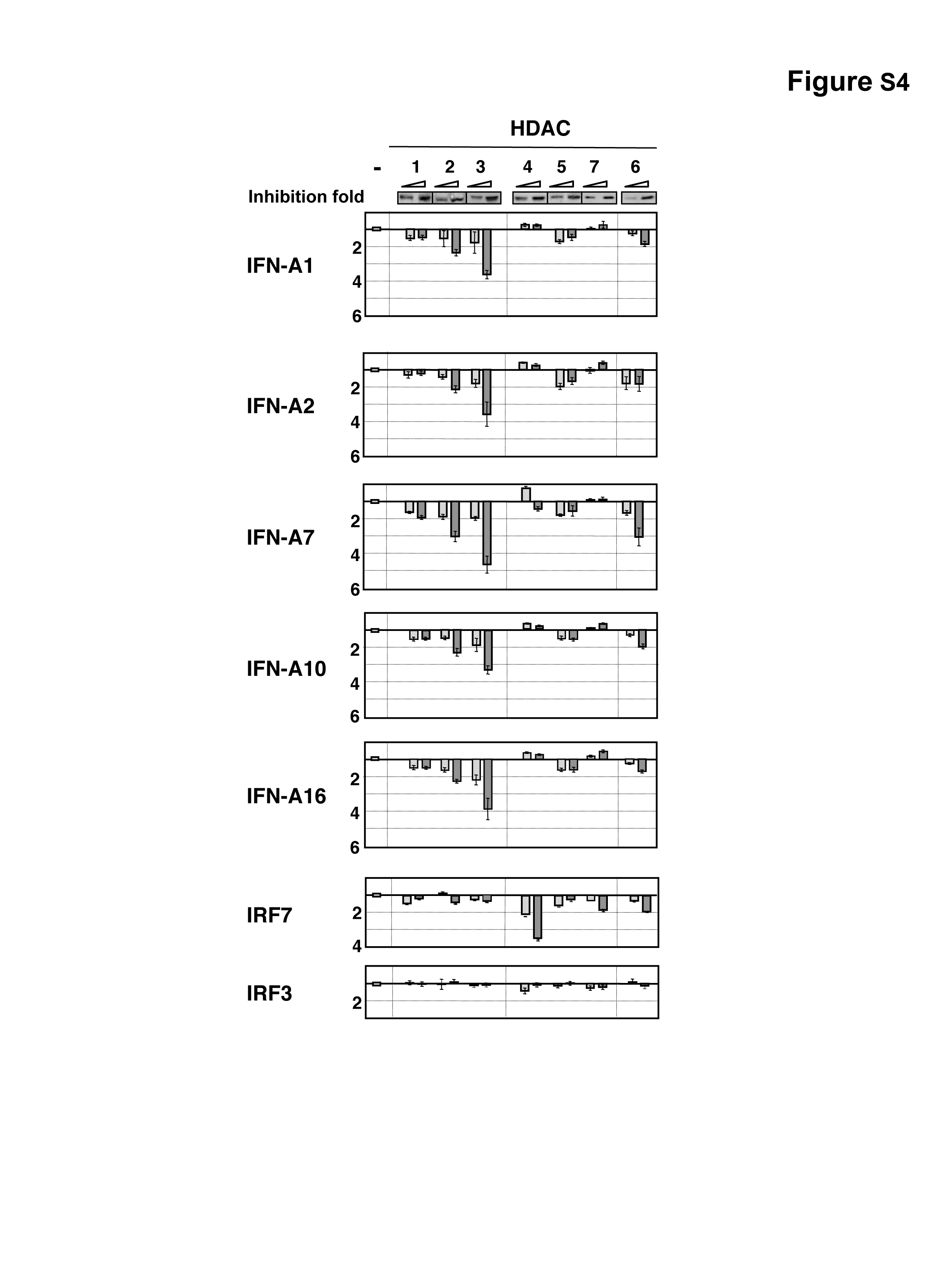

Supplement: Figure S4 — Effect of HDAC overexpression on IRF7-mediated IFN-A gene transcription. The effect of HDACs class I (1, 2 and 3), class IIa (4, 5, and 7) and class IIb (6 and 10) on IFN-A and IRF gene transcription was determined in HEK293-TLR3 cells transfected with pcDNA3-IRF7A together with an HDAC-encoding plasmid added in 2-fold increasing amounts. After 24 h of expression, the inhibition fold was calculated from IFN-A mRNA levels determined by RT-QPCR in HDAC-expressing cells in comparison to control cells transfected with pcDNA3. Expression levels of each HDAC determined by anti-flag immunoblotting of the cell lysates are shown in the insets. (TIF) [file pone.0038336.s004.tif]
